# Editorial: Advances in smart bioactive surfaces for wounding healing and tissue repair

**DOI:** 10.3389/fbioe.2023.1140176

**Published:** 2023-01-18

**Authors:** Changjiang Pan, Tao Liu, Ying Yang

**Affiliations:** ^1^ Faculty of Mechanical and Material Engineering, Jiangsu Provincial Engineering Research Center for Biomaterials and Advanced Medical Devices, Huaiyin Institute of Technology, Huaian, China; ^2^ Medical College of Acu-Moxi and Rehabilitation, Guangzhou University of Chinese Medicine, Guangzhou, China; ^3^ Cardiac Surgery Department, Michigan Medicine, University of Michigan, Ann Arbor, MI, United States

**Keywords:** bioactive surface, wounding healing, tissue repair, medical devices, biomaterials

Biomaterials and implantable/interventional medical devices play an irreplaceable role in saving human life and promoting human health. Their surface properties and functions largely determine their clinic performances after the implantation. The introduction of the bioactive factors on the surfaces to design the surface biological function and construct the surface microenvironment, so as to further endow the surfaces with the ability to *in situ* regulate the physiological microenvironment responses, can effectively promote wound healing and tissue repair and regeneration after the implantation. In particular, the smart bioactive surfaces on the implants, which can not only mimics the *in vivo* three-dimensional microenvironment but also promote wound healing and tissue repair through the *in vivo* physiological environment, can endowed the implant with the multi-functional ability to actively adapt to and regulate the interfacial physiological responses, resulting in better promotion of wound healing and tissue repair.

The Research Topic mainly focuses on the most recent *advances in smart bioactive surfaces for wounding healing and tissue repair*, which can be potentially used in the clinic biomaterials and implants. We have collected two review articles and two original research articles, which highlight several emerging trends of smart bioactive surfaces for wounding healing and tissue repair.

Xia et al., reviewed the application of chitosan-based materials in surgical or postoperative hemostasis. In this review, the physiological hemostasis, the physicochemical properties of chitosan and its role in hemostasis were firstly discussed in brief. Subsequently, the recent advances in the chitosan-based hemostatic materials, including hydrogels, dressing, sponge and nanocomposites, were mainly summarized in detail. The methods to increase the water absorption, porosity, and compressive strength of the chitosan-based hemostatic material are also discussed for each kind of chitosan-based hemostatic materials. Finally, the authors emphasized that, although chitosan-based hemostatic materials have shown good effects *in vitro* and *in vivo* animal experiments, they have not been widely in clinic. Due to the advantages of higher hemostatic efficiency and lower price of chitosan-based hemostatic materials than the existing hemostatic materials such as gelatin sponge, they will have important roles in clinical practice, which is worth further strengthening exploration.

Hydrogels are the attractive candidates as fillers for bone injuries with irregular shapes and as carriers for local therapeutic treatments, such as osteoporosis. In the review article by Gong et al., the hydrogel-based delivery system applied in the local anti-osteoporotic bone defects was summarized. The hydrogels for adjuvants loading, including nature-based, synthetic, and composite hydrogels, were firstly reviewed, followed by discussing the anti-osteoporosis adjuvants-loaded hydrogel for preventing osteoblast and promoting osteogenesis. The hormone analogs and osteo immunomodulators loaded in the hydrogels for osteoporosis treatment was also discussed briefly. Finally, the authors pointed out that the detection of the ideal hydrogel-adjuvant system for the treatment of the local anti-osteoporotic bone defects had the potential to ease the transition from basic research to clinical application, and it required the joint efforts of clinicians and researchers.

In the article by Zhang et al., the biomechanical and clinical properties of the novel internal fixation Interlocking Hip Screw (IHS) and conventional inverted triangle cannulated screws (ITCS) for treatment of Pauwels III femoral neck fractures were comparatively studied. Twenty synthetic femurs were osteotomized to simulate 70° Pauwels Ⅲ femoral neck fractures and randomly divided into two groups: Group IHS and Group ITCS. The biomechanical and the clinical properties, such as demographic data, operating duration, intraoperative blood loss, number of fluoroscopies, length of hospital stay, fracture healing time, Harris Hip Score (HHS), the score of Visual Analogue Scale (VAS) and complications such as non-union, avascular necrosis, and femoral neck shortening were comparatively explored. The results indicated that IHS provided better biomechanical and clinical performances due to its unique biological and biomechanical mechanisms, compared with ITCS. Thus, the author believed that IHS was a feasible alternative to ITCS for the fixation of Pauwels Ⅲ femoral neck fractures.

Insufficient oxygen supply and hypoxia caused by tumor lead to a poor therapeutic effect and poor prognosis. In order to overcome this issue, Wang et al., prepared a kind of nanoparticles called W18O49@EP to release reactive oxygen species (ROS) during the combined tumor radiotherapy (RT) and photodynamic therapy (PDT). It was found that the ROS release by the nanoparticles during near infrared light (NIR) irradiation can enhance the effect of PDT treatment without inducing hypoxia. Hence, this strategy can greatly improve the anti-tumor effect, which could provide an effective approach for clinical anti-tumor studies.

Overall, this Research Topic covers recent advances in smart bioactive surfaces for wound healing and tissue repair. The editors hope that the current Research Topic will contribute to the research and development in the field of surface modification of biomaterials and implantable/interventional medical devices for wounding healing and tissue repair, inspiring future exploration to expand the biomedical applications of smart bioactive biomaterials.

